# Trans Oral Endoscopic Thyroidectomy Vestibular Approach (TOETVA) in Brazil: Safety and complications during learning curve

**DOI:** 10.20945/2359-3997000000380

**Published:** 2021-06-29

**Authors:** Antonio Augusto Tupinambá Bertelli, Leonardo Guimarães Rangel, Renan Bezerra Lira, Marco Antonio Scirea Tesseroli, Izabella Costa Santos, Guilherme Duque Silva, Michelle Azevedo Gomes, Lucas Ribeiro Tenório, Luiz Paulo Kowalski, Antonio José Gonçalves, Jonathon Owen Russel, Ralph Patrick Tufano

**Affiliations:** 1 Faculdade de Ciências Médicas da Santa Casa de São Paulo Departamento de Cirurgia São Paulo SP Brasil Disciplina de Cirurgia de Cabeça e Pescoço, Departamento de Cirurgia, Faculdade de Ciências Médicas da Santa Casa de São Paulo, São Paulo, SP, Brasil.; 2 Universidade do Estado do Rio de Janeiro RJ Brasil Universidade do Estado do Rio de Janeiro, RJ, Brasil.; 3 A.C. Camargo Cancer Center São Paulo SP Brasil A.C. Camargo Cancer Center, São Paulo, SP, Brasil.; 4 Unimed Chapecó Hospital Chapecó SC Brasil Unimed Chapecó Hospital, Chapecó, SC, Brasil.; 5 Instituto Nacional do Câncer Rio de Janeiro RJ Brasil Instituto Nacional do Câncer, Rio de Janeiro, RJ, Brasil.; 6 Universidade do Estado do Rio de Janeiro Hospital Central da Polícia Militar do Rio de Janeiro Rio de Janeiro RJ Brasil Serviço de Cirurgia de Cabeça e Pescoço, Hospital Central da Polícia Militar do Rio de Janeiro, Universidade do Estado do Rio de Janeiro, RJ, Brasil.; 7 Universidade do Estado do Rio de Janeiro Hospital Federal de Bonsucesso RJ Brasil Hospital Federal de Bonsucesso e Universidade do Estado do Rio de Janeiro, RJ, Brasil.; 8 Johns Hopkins Medicine Baltimore USA Head and Neck Endocrine Surgery, Otolaryngology-Head and Neck Surgery, Johns Hopkins Medicine, Baltimore, USA.

**Keywords:** Thyroid cancer, goiter, surgery, thyroidectomy, endoscopic

## Abstract

**Background:**

The aim of this study was to address the first cases of TOETVA done in Brazil, by TOETVA-Bra study group, regarding safety and complications.

**Materials and Methods:**

Series of the first 93 TOETVAs cases in Brazil. All authors except LPK, AJG JOR and RPT received TOETVA training including cadaveric hands-on in Thailand or United States (Johns Hopkins Medicine) during 2017. After they came back to Brazil and started doing their first TOETVA cases in the cities of Rio de Janeiro, Sao Paulo and Chapecó they agreed to collaborate and gather data using an online spreadsheet. All patients were submitted to the technique described by Anuwong.

**Results:**

A total of 93 patients underwent TOETVA. Most patients (58.1%) were submitted to total thyroidectomy and 59.1% had benign disease. Two patients (2.2%) needed conversion to open surgery. Five patients (9.3%) developed transient hypoparathyroidism and there were 3 (2.0%) temporary recurrent laryngeal nerve palsy. There was one (0.7%) permanent unilateral palsy. Twenty patients had some sort of complication, 16.1% were minor and 5.4% were major. A total of 73 patients (78.5%) had an uneventful recovery.

**Conclusion:**

The technique is reproducible with a low complication rate. While further studies are needed to confirm equivalency, early efforts suggest that TOETVA is not inferior to traditional open thyroidectomy in appropriately selected patients.

## INTRODUCTION

This decade has brought many modifications to the standard open thyroid surgery technique (1). From the beginning of remote access thyroidectomy, when the techniques focused on hiding the scar in more discrete locations (2-4), until now, scarless thyroid and central neck surgery has evolved and may be safely completed via the transoral vestibular approach (1,5–8).

The Transoral Endoscopic Thyroidectomy Vestibular Approach (TOETVA) is the first remote access technique of thyroid surgery to become widely popular in the Western Hemisphere (9-13). Before this procedure, a few surgeons were performing other minimally invasive techniques such as minimally invasive video-assisted thyroidectomy (MIVAT) or axillary, breast or post-auricular approaches, but without reproducible results (2,3,14). These techniques were not widely adopted for a number of reasons (15). The aim to avoid a neck scar was the fuel to develop, improve and spread remote access thyroid surgery. Anuwong and cols., in 2016, published a series of the first 60 human cases with good results (16). The technique was adopted by some US head and neck surgery centers (9).

This article was planned by a team of Brazilian surgeons trained in TOETVA in the United States and Thailand. Each one did the training program individually and founded the TOETVA-Bra study group aiming to collect data from the first Brazilian cases. All Brazilian authors work in high volume head and neck surgery centers that perform more than 50 thyroidectomies per year.

The aim of this study was to address the first cases of TOETVA performed in Brazil, by TOETVA-Bra study group, with a specific focus on safety and outcomes of adoption in high volume centers.

## MATERIALS AND METHODS

All authors except LPK, AJG, JOR and RPT received TOETVA training including cadaveric hands-on in Thailand or United States (Johns Hopkins Medicine) during 2017. After they came back to Brazil and started doing their first TOETVA cases in the cities of Rio de Janeiro, Sao Paulo and Chapecó. They agreed to collaborate and gather data using an online spreadsheet shared with Numbers^®^ (Apple inc). RT and JOR are Head and Neck Surgeons experienced with TOETVA who participated in the authors training. This study has IRB approval from all the institutions collecting data: Santa Casa de São Paulo Faculty of Medical Sciences – main institution (3.897.377) (CAAE: 27131119.2.0000.5479), A.C. Camargo Cancer Center (1913/14), Federal Hospital of Bonsucesso (2.825.617), Brazilian National Cancer Institute (89042418.7.0000.5274), State University of Rio de Janeiro (07678819.0.0000.5259), and Unimed Chapecó Hospital (089/2018).

The surgical technique employed was that described by Anuwong in 2016 (1) using 3 endoscopic ports through the oral vestibule, dissecting a subplatysmal pouch after hydrodissection and dilation, insuflating this pocket with high flow and low pressure CO2, opening the raphe to identify and divide the isthmus, and resecting the thyroid gland. It also involves identifying both parathyroid glands and the recurrent laryngeal nerve on each side using conventional laparoscopic instruments. Nerve monitoring (Neurosoft, Ivanovo, Russia or Medtronic, Dublin, Ireland) was used in all surgeries to help visual identification and preservation of the recurrent laryngeal nerve. In some cases, the nerve monitor was also used for superior laryngeal nerve preservation and vagal stimulation. Ultrasonic or advanced bipolar laparoscopic devices (Johnson & Johnson, New Jersey, USA or Medtronic, Dublin, Ireland, respectively) were used to seal thyroid vessels in all cases. The specimen was always taken out with an endoscopic bag through the midline incision. All procedures were done under general anesthesia.

The data included was demographic information, size of dominant nodule, Fine Needle Aspiration Cytology (FNAC) result, extension of surgery, need for conversion to open surgery, final pathology results and occurrence of complications during the first 30 postoperative days. Complications were divided in minor and major. Minor complications were temporary events without sequelae and major complications were considered permanent events with some sequelae or events that threaten life or may need hospitalization.

## RESULTS

A total of 93 patients underwent TOETVA from June 2017 to January 2019. Of these, 79 (85.0%) were women and 14 were men. It was estimated the participation of 40 surgeons in all procedures. The total number of leading surgeons was 7 (Table 1). As the procedures occurred in a high number of different hospitals in Brazil, including teaching institutions with residents, it wasn’t possible to determinate the exact number of doctors involved in these surgeries. Most patients (n = 54, 58.1%) were submitted to total thyroidectomy and 39 (41.9%) to lobectomy (Table 2). Median age of patients was 41 years (ranging from 15 to 69 years). Median nodule size was 1.8 cm (ranging from 0.6 cm to 6.0 cm). A total of 55 (59.1%) patients had benign disease and 38 (40.8%) had malignant disease, mostly T1 (32 cases) and six T2 papillary carcinomas.

**Table 1:** Number of procedures for each leading surgeon

Surgeon	n
LGR (RJ)	34
RBL (SP)	20
MAST (SC)	11
AATB (SP)	8
GDS (RJ)	8
MAG (RJ)	8
ICS (RJ)	4
Total	93

**Table 2:** Extension of thyroidectomy performed

	Benign	%	Malignant	%	Total	%
TT	24	44	30	79	54	58
Lobectomy	31	56	8	21	39	42
Total	55	100	38	100	93	100

TT: total thyroidectomy.

Indications for surgery based on Bethesda classification and nodule size are shown in Table 3. Two patients (2.2%) needed conversion to open surgery because of bleeding with no other complications reported.

**Table 3:** Indications for surgery based on Bethesda Fine Needle Aspiration Biopsy (FNAB), corresponding pathology report and median nodule size

FNAB (Bethesda)	n	Pathology report	Median nodule size (cm)
I	1	Benign	
II	14	All benign	4,0
III	21	4 malignant (19%)	2,4
IV	21	5 malignant (24%)	1,8
V	16	13 malignant (81%)/3 NIFTP*	1,5
VI	15	All malignant (100%)	1,0
Ignored	5	1 malignant, 4 benign	
Total	93		

* NIFTP: Noninvasive follicular thyroid neoplasm with papillary-like nuclear features.

**Figura 1:**
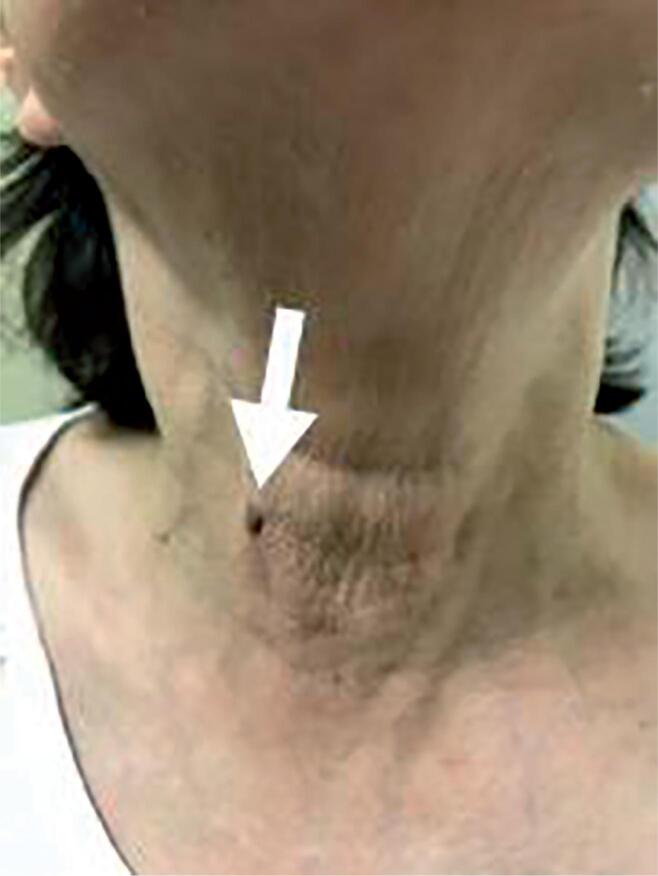
Example of skin burn (white arrow) due to space creation using hook monopolar device.

Complications are shown in Table 4. Considering 54 total thyroidectomies, 5 patients (9.3%) developed transient hypoparathyroidism. There was no permanent hypoparathyroidism. Considering 147 nerves at risk, there were 3 (2.0%) temporary recurrent laryngeal nerve palsy (all have function returned after 3 months). There was one (0.7%) permanent unilateral palsy. No laryngeal nerves were known to be severed during this study. Three patients (3.2%) had small skin burns (Figure 1). Two patients (2.1%) had suture dehiscence of central incision that healed spontaneously after two weeks. One patient (1%) had surgical site infection treated with needle aspiration and antibiotics with no need for reoperation. One patient (1%) had a small tracheal tear (2 mm). This lesion was identified intraoperatively and endoscopically sutured after removing the thyroid.

**Table 4:** Complication rates in TOETVA

Complications	n	%	Note
No	73	78.5	
Yes	20	21.5	
Transient hypopara	5	9.3	54 TT
Transient RLN palsy	3	2.0	147 nerves at risk
Permanent RLN palsy	1	0,7	147 nerves at risk
Skin burn	3	3.2	
Mental nerve palsy	1	1.1	
Infection	1	1.1	
Bleeding/conversion	2	2.2	
Suture dehiscence	2	2.2	
Tracheal lesion	1	1.1	
Mandibular palsy	1	1.1	

TT: total thyroidectomy.

The patient healed well after one week with a suction drain. One patient had a temporary unilateral marginal mandibular nerve palsy for 3 months. It was identified a minor lip weakness in the first day post-operatively and the patient evolved with full spontaneous recovery. One patient (1%) had transient mental region anesthesia that was resolved after 5 months. There was no hematoma in this series.

Considering lobectomies and total thyroidectomies (93 cases), 20 had some sort of complication (21.5%), 16.1% were minor complications (transient hypoparathyroidism, transient recurrent laryngeal nerve palsy, skin burn, suture dehiscence, mental nerve and marginal mandibular nerve transient palsy) and 5.4% were major complications (permanent vocal fold palsy, permanent hypoparathyroidism, surgical site infection, tracheal perforation and conversion). A total of 73 patients (78.5%) had an uneventful recovery.

## DISCUSSION

This study represents the initial experience of Brazilian surgeons with a new thyroid surgery technique – TOETVA. All surgeons involved in the 93 cases are pioneers in this new procedure in Brazil. This technique has evolved in eastern countries such as Thailand and South Korea due to a common goal to avoid the visible scar and also optimize access to the central neck (1,17,18). It has brought better cosmetic results and quality of life according to some authors in the earliest series of cases (1,19).

Although other minimally-invasive techniques for thyroid surgery were performed before in Brazil, TOETVA is the first one to reach a high number of surgeons. It is probably associated with better acceptance by Brazilian surgeons due to its wide operative indications (20), reproducibility and its lower cost. The instrumentation is also widely available, as it requires only standard laparoscopic instruments (1).

The beginning of the learning curve is tricky mostly because is hard to choose the first cases. Although TOETVA shows itself as a technique with wide operative indications, surgeons take some time to get used to it. Perform some and learn with more experienced colleagues drives the surgeon to improve skills and start to indicate more. Commonly the learning curve starts with single small benign nodules, treated with lobectomies. Since the daily main routine of Head and Neck surgeons in Brazil are thyroid nodules, its guaranteed that we still perform a much higher number of conventional thyroidectomies.

Of note, our series showed that most patients (N = 54, 58.1%) underwent total thyroidectomy and 40% were performed for malignant disease. This suggests that Brazilian surgeons felt comfortable progressing quickly from the indications for benign to malignant disease. The low median age (41 years old) is consistent with our anecdotal experience that young patients usually show more interest in TOETVA. The low median nodule size (1.8 cm) demonstrates that patient selection was followed by the surgeons, although some 6 cm (benign) nodules were also addressed. In those patients who had benign FNAC findings, the median nodule size was higher (4.0 cm), similar to traditional indications for thyroidectomy in benign nodules.

The sample is in accordance with the indications determined by the literature, being a good indication for TOETVA: thyroids up to 10 cm wide, benign nodules up to 6 cm and malignant nodules up to 2 cm (1). As the working space has its limitations and the specimen removal should be performed trough the central inferior lip incision the nodule size and the thyroid width are the most limiting aspects of the technique. Fragmentation of the thyroid is reported and other incisions are been used in some centers to remove large specimens, however, these doesn’t solve the limited space issue and fragmentation of nodules are not recommended.

Despite the aesthetic appeal, it is a new technique in Brazil and in the first cases it was necessary to present the procedure to patients, however, it has become increasingly common to seek the technique as it has been consolidated. Surprisingly 15% of patients are male, confronting the belief that good cosmetic results in this population are not relevant. As these patients were not the first cases, the vast majority sought the technique on their own.

Only 2 patients needed conversion to open surgery due to bleeding and it occurred in the first cases. In one, arterial bleeding started when the surgeon was ligating the superior pole and in the other the bleeding emerged from the thyroid and the leaking spot was not clearly identified. We believe that the magnification of the image and the video equipment can induce the surgeon to overestimate the bleeding. We also think that the lack of expertise with instruments and the laparoscopic technique, specially the 2D view, were major factors for the conversion in these cases.

The complication rate of TOETVA was very similar to the complication rates of the traditional open technique described by other authors (1,16,18,21,22). This series presented a 9.2% rate of transient hypoparathyroidism with no cases of permanent hypoparathyroidism. The rates of transient and permanent inferior laryngeal nerve palsy were similar to other studies of TOETVA (1,16,17,18,21,22). It is likely that there is a selection bias due to cases of benign disease, predominantly small nodules, and carefully selected early stage cancer, which could all be associated with improved outcomes when compared to series of traditional open thyroidectomy with broader operative indications (23). It should be noted that the video magnification and smoothness of dissection due to small and delicate instrumentals add safety to recurrent laryngeal nerve dissection, especially after learning curve has ended, although it is not clear if this will result in an improvement over the excellent safety profile in traditional thyroid surgery. Future studies will help to elucidate this point.

Every new procedure brings new complications. This is especially true with remote access surgery, where development of a working space can introduce novel complications relevant to the local anatomy. With TOETVA, this anatomy stretches from the oral vestibule to the central neck and complications may include (17) skin burns, hypoesthesia due to mental nerve trauma, marginal mandibular nerve palsies and neck infection. The skin burns occurred because an energy device was used close to the skin, usually a monopolar hook. These complications generally occurred early in the surgeon’s operative experience, and they generally healed well (24). Hypoesthesia due to mental nerve trauma was temporary and occurred only once, during the first cases, when we were still placing the incisions closer to the mandible. Modifications were made, and all surgeons started placing the central incision closer to the lip and the lateral incisions closer to the oral commissure. The group decided to do this modifications after conversations with more experienced surgeons in the US and Thailand, who observed that the lower incisions were closer to mental nerve foramen. Since that time, there have been no more mental nerve injuries (1,17,24). The marginal mandibular nerve palsy is interesting, as it is not common in other series. It may have occurred due to lip musculature being traumatized during placement of the lateral trochar rather than a true marginal mandibular nerve weakness. Although it could be explained by incorrect placement of lateral trochars, connected to some anatomic variation, this seems less likely due to the position of the ports and the likelihood that any injury would be to no more than a terminal branch of the marginal mandibular nerve. Possibly, the use of permanent metallic trochars contributed, as they may be more likely to permit energy conduction. These trochars are frequently used in Brazil due to cost savings. Wound infection isn’t common in traditional open surgery, and neither is it common in TOETVA as shown by other authors (16-18). The patient who had an infection recovered promptly with antibiotic treatment with amoxicillin with clavulanate and needle aspiration. Bacterial cultures were negative. One tracheal lesion occurred which is also a possible complication in open surgery. In this situation, however, the corresponding surgeon believes that the decreased tactile sensation during endoscopic surgery contributed to this injury. Finally, there were two episodes of suture dehiscence in the mouth that were not treated with any intervention. No long term effects were noted in those patients.

More recently novel technique without CO2 insufflation was developed based on TOETVA (25). It is important to differentiate both procedures, since TOETVA is getting popular worldwide and day by day more surgeons are starting to do it. The CO2 free technique is still in progress. A few number of surgeons are performing it and additional specific instrumentation are needed (25). Although good results are observed without CO2 insufflation, this results are still note reproducible in a worldwide scale and more data is needed.

TOETVA is a feasible and safe procedure in high volume thyroidectomy centers of Brazil (26,27). It is reproducible and the learning curve is around 10-15 cases (1,17,24,26,28). It seems to be spreading quickly and reaching many Brazilian surgeons because of excellent cosmetic results. Cultural factors and patient demand will likely continue to drive requests for the procedure. More experience in this field should bring more applicability and innovation to the technique and further studies should focus on advanced procedures such as central neck dissection. TOETVA is a novel method of performing central neck surgery without a cutaneous incision that is becoming more popular and has demonstrated good outcomes thus far in Brazil.

In conclusion, the first 93 cases of TOETVA reported in Brazil demonstrates a complication rate similar to that of traditional open thyroidectomy. Its adoption is feasible and safe in high volume centers. More experience with this technique should bring more applicability of the endoscopic central neck vestibular approach technique.
